# On the Use of Clay in Surgery

**Published:** 1907-02-23

**Authors:** John Aikman

**Affiliations:** Honorary Surgeon, Colonel and Principal Medical Officer, Royal Guernsey Militia; late Surgeon, St. Peter's Port Hospital


### GENERAL PRACTIONERS' COLUMN.

### ON THE USE OF CLAY IN SURGERY.

By JOHN AIKMAN, M.D.Glasg., Honorary Surgeon, Colonel and Principal Medical Officer, Royal Guernsey Militia; late Surgeon, St. Peter's Port Hospital.

THE United States Pharmacopœia has officially recognised clay in the form of a poultice, and general practitioners in this country are using antiphlogistine as an external application to inflammations unattended by breach of surface. We believe therefore the following interesting contribution from Dr. Aikman, of Guernsey, must prove of practical value in many ways. Dr. Aikman writes: At the outset of the antiseptic system I had the privilege of being a dresser to Lord Lister. Carbolic acid was at that time (1866-7) very impure and did not dissolve readily in water; some of its impurities remained undiluted and had a caustic action on the skin. It occurred to Lord Lister to make a more complete solution in linseed oil, but this was found inconvenient for use. An endeavour was then made to incorporate the carbolised oil with whiting in the form of putty, and some very good results were obtained from the putty, especially in cases of compound fractures and contused wounds. The results in psoas abscess were less favourable. The oily solutions had a strength of 1 in 4, 1 in 7, 1 in 10, 1 in 20, and 1 in 40. In January 1868 the present writer pointed out that the amount of carbolic acid in the putty (which required 5 or 6 parts of whiting for each part of oil) was far below the standard recognised as a reliable antiseptic, and that some other explanation of its authenticated results in compound fracture must be forthcoming. Lord Lister took the suggestion to avizandum and abandoned the putty. The shellac dressing followed, but its principles are outside the immediate subject. Of recent years, by the use of gauze covered by various wools, aseptic or antiseptic, there has been a return to the quietude which the putty secured. The fact is that the putty, or the gauze and wool dressing, secure rest of the parts and favour the tissues in their struggle to overcome septic invasions when the injured parts can be controlled and kept free from movement by the splint-like action of the dressing. That condition is not present in the case of psoas abscess. Once more it was noticed that the putty became wet, while the skin remained greasy from an exudation of the antiseptic oil from the putty, and the oil held the antiseptic firmly.

Reviewing these observations from the standpoint of the uses of antiphlogistine, and comparing them with some considerable experience of this preparation, I would place in the first rank its use in keeping at rest, and free from infection from the skin, breasts which it is desirable to rid of milk. Put the antiphylogistine on thick, and cover with cotton-wool supported by a bandage. In a day or little more, the breasts are normal if the infant is kept out of the mother's sight. The same condition of rest favours the treatment of boils and carbuncles. Pleuritic effusion is controlled on the same principle, and with the advantage over strapping that the dressing can be removed for the daily examination of the chest. In synovitis of the knee, even with fracture of the patella, the closely-moulded dressing secures additional rest to that afforded by a splint, and it may be left on for a considerable time. I have also used it in inflamed glands. The salicylic acid in the oil of wintergreen is a good antiseptic in cases in which the skin is fairly normal, and does not irritate. Of the other uses of antiphlogistine I have nothing to say; some of those claimed seem to me to be better met by more strenuous means.

